# Ranibizumab for the treatment of retinopathy of prematurity: systematic review and meta-analysis

**DOI:** 10.3389/fped.2023.1202927

**Published:** 2023-08-04

**Authors:** Niza Alva, Alex R. Martínez, Brando Ortiz-Saavedra, Elizbet S. Montes-Madariaga, Alonso Cotrina, José A. Caballero-Alvarado, Ranjit Sah, Joshuan J. Barboza

**Affiliations:** ^1^ Facultad de Medicina, Universidad Peruana de Ciencias Aplicadas, Lima, Peru; ^2^ Unidad de Revisiones Sistemáticas y Meta-Análisis, Tau-Relaped Group, Lima, Peru; ^3^ Facultad de Medicina, Universidad Nacional de San Agustín de Arequipa, Arequipa, Peru; ^4^ Facultad de Medicina, Universidad Peruana Cayetano Heredia, Lima, Peru; ^5^ Facultad de Medicina, Universidad Privada Antenor Orrego, Trujillo, Peru; ^6^ Institute of Medicine, Tribhuvan University Teaching Hospital, Kathmandu, Nepal; ^7^ Department of Microbiology, Dr. D. Y. Patil Medical College, Hospital and Research Centre, Dr. D. Y. Patil Vidyapeeth, Pune, India; ^8^ Department of Public Health Dentistry, Dr. D.Y. Patil Dental College and Hospital, Dr. D.Y. Patil Vidyapeeth, Pune, India; ^9^ Escuela de Medicina, Universidad Cesar Vallejo, Trujillo, Peru

**Keywords:** retinopathy of prematurity, systematic review, ranibizumab, meta-analysis, laser therapy

## Abstract

Anti-VEGF drugs like ranibizumab can be used to treat retinopathy of prematurity (ROP) by halting the formation of abnormal blood vessels, or lasers can be used to burn the edges of the retina where these vessels are present. The objective is to compare the efficacy for ROP between ranibizumab and laser therapy.

**Material and methods:**

Electronic searches will be carried out in medical databases with key words and controlled vocabulary terms. Randomized controlled trials (RCT) will be assessed. The primary outcome will be the full ROP regression. Two reviewers will extract the data using predefined forms and, to assess the quality of the study, we will use RoB 2.0, the tool for randomized controlled trials developed by the Cochrane Collaboration. We used a combination of the inverse-variance approach and random-effects models for the meta-analysis.

**Results:**

The eyes of 182 preterm infants who had ranibizumab treatment were assessed in a total of 364 eyes, and 135 infants received laser therapy. The follow-up period was between 6 and 24 months. Ranibizumab was not associated with greater regression of ROP compared to laser therapy in preterm infants (RR: 1.09, CI 95%: 0.95–1.24; *p*: 0.22). Also, ranibizumab was not associated with recurrence of ROP compared to laser therapy in preterm infants (RR: 3.77, CI 95%: 0.55–25.81; *p*: 0.22).

**Conclusions:**

The efficacy of ranibizumab compared to laser is very uncertain in terms of ROP regression and decreased ROP recurrence in preterm infants.

**Systematic Review Registration:**

identifier PROSPERO (CRD42022324150).

## 1. Introduction

Due to the growth of abnormal blood vessels in the retina, retinopathy of prematurity (ROP), a developmental condition in preterm neonates before 31 weeks, can cause low visual acuity or juvenile blindness (1). This condition can be treated with intravitreal injections to stop these vessel growth or laser photocoagulation to ablate the aberrant vascularization and prevent further damage (2). This condition has also been linked to infant morbidities such as sepsis, intraventricular bleeding, respiratory distress syndrome, and low weight gain (3).

By 36–40 weeks of gestation, the normal vessel proliferation is complete, starting around 12 weeks. Prior to that, the optic disc is where normal proliferation begins, before moving on to the ora serrata (4). The retina of newborns is underdeveloped and has an avascular zone. The pathology has two phases: first, it has an avascular zone owing to hyperoxia or fluctuating oxygen levels, and second, the avascular zone of retina results in a high concentration of vascular endothelial growth factor (VEGF), which promotes the formation of abnormal vasculature (5). The medication used for intravitreal injections include ranibizumab and bevacizumab (6), which are anti-VEGF, both have shown low prevalence of developing refractive errors in the future although bevacizumab-treated eyes in severe cases of ROP were linked to a higher likelihood of myopia or neurodevelopmental damage (7).

Currently, anti-VEGF agents are not approved by the Food and Drug Administration (FDA) for the treatment of ROP (8, 9). However, current consensus ROP screening guidelines published by the American Academy of Pediatrics in conjunction with the American Academy of Ophthalmology recommend the use of intravitreal anti-VEGF as a promising treatment for ROP type 1 and other types of ROP (10). In recent clinical trials ranibizumab was revealed to be an effective and safe treatment compared to laser therapy. Despite the fact that it is linked to higher immediate results and lower risk of unfavorable structural changes, it is important to consider the risk of complications that can be proper of the procedure and also the necessity of constant subsequent examinations to prevent late reactivation of ROP or long-term complications.

This systematic review assessed all of the known controlled studies of ranibizumab compared with laser photocoagulation. The aim is to compare the efficacy for ROP between ranibizumab and laser therapy.

## 2. Materials and methods

### 2.1. Search of studies

Electronic searches were carried out in EMBASE, Pubmed, Web of Science (WOS) and Scopus. Keywords and controlled vocabulary terms [i.e., medical subheading (MeSH) terms EMTREE terms] included patient-related terms (premature, retinopathy of prematurity) and treatment-related terms (ranibizumab). There was not a limit by year of publication. Two writers conducted the search. This study was reported following the recommendations of PRISMA-2020 guidelines.

### 2.2. Eligibility criteria

Randomized controlled trials (RCTs) and nonrandomized intervention studies (NRIS) in preterm newborns (<31 weeks of gestational age), studying ranibizumab administration to manage ROP were included but only RCTs comparing ranibizumab use with laser photocoagulation were considered for meta-analysis. We excluded case reports, editorials, narrative reviews, observational studies, and other systematic reviews.

### 2.3. Search of studies

Two reviewers extracted data using predefined forms. After eliminating duplicates, two independent reviewers examined each article by first considering only the title and abstract of the study, and then two independent reviewers evaluated the full text of the initially included studies.

### 2.4. Outcomes

We established as the primary outcome the complete regression of ROP and the ones defined as secondaries recurrence of ROP, change of treatment, refractive error, and adverse events (retinal or vitreous hemorrhage, endophthalmitis, conjunctivitis, corneal opacity, cataract, or other complications).

### 2.5. Data extraction and management

Three reviewers independently extracted relevant data from those included in a pre-established electronic sheet and conflicts were resolved jointly by consensus. The data to evaluate was: principal author, year of publication, clinical trial design, clinical trial phase, country(ies) involved in the study, number of individuals per arm, characteristics of the intervention (ranibizumab) and of the comparison, primary (complete ROP regression) and secondary outcomes.

### 2.6. Evaluation of the quality

The Cochrane Collaboration tool for randomized controlled trials (RoB 2.0) was utilized for conducting the quality assessment.

### 2.7. Strategy for data synthesis

We selected the random-effects model and inverse variance method to perform the meta-analysis of our established outcomes (11). Similar to other previous studies applied (12), we evaluated the Paule-Mandel estimator to assess between-study variance (13). The effects of ranibizumab on continuous outcomes were expressed as mean difference (MD) with 95% confidence intervals (95% CI). For dichotomous outcomes, we assessed relative risk (RR) with CI 95%. For each trial arm the baseline values for continuing outcomes have been adjusted. *I*^2^ statistic was used to regulate the statistical heterogeneity of effects in the RCTs as it was used in other meta-analyses (14–16), which had 3 categories for the level of heterogeneity: high (>60%), medium (30%–60%), and low (<30%) (17).

The model for analyzing the sensitivity in this meta-analysis was Fixed-effects, and for the methods, the Mantel-Haenszel method for the primary outcomes. In this article, we used the metacont and metabin functions of the meta package of the R 3.5.1 software. In addition to this, to evaluate the quality of evidence (QoE) we utilize the GRADE approach (18). We appraised imprecision, publication bias, risk of bias, quality of evidence and inconsistency among studies. We execute confidence ratings as very low, low, moderate and high. In SoF (summary of results) tables we specified QoE and at the end, we created the SoF tables applying GRADEpro GDT.

## 3. Results

### 3.1. Study selection

**Figure 1:**
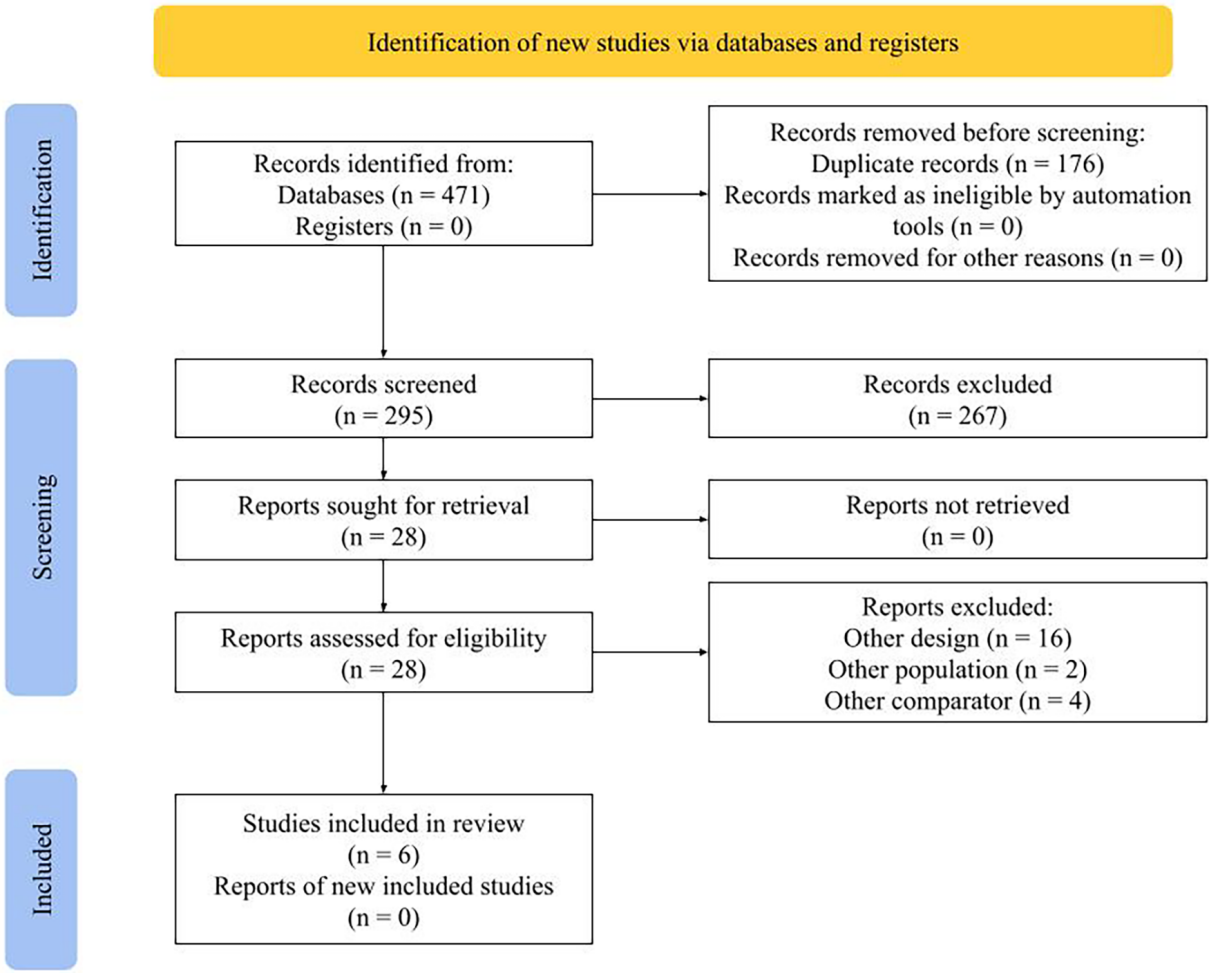
PRISMA-2020 flowchart of study selection.

The method of searching retrieved a total of 471 items. The PRISMA flow chart displays the selection methodology (PRISMA 2020) (19). We reviewed 295 articles after removing duplicates which were 176 in total. Subsequent to an exhaustive review of the titles and abstracts, we chose 28 articles for a full-text read; from them, 6 were deemed suitable for systematic review (20–25) (Figure 1).

### 3.2. Selected studies aspects

Altogether, 364 eyes of 182 preterm newborns treated with ranibizumab were evaluated, and 135 newborns were treated with laser. The mean of gestational age was 26.7 weeks (SD: 1.8). We included two open label RCT's (21, 22). One trial (RAINBOW study) reported results at 6 months (22, 25) and 24 months (23, 24) in 4 articles. Likewise, we founded one NRSI open label study (20). The follow-up time was ranged 6–24 months. The population considered between studies were preterm newborns diagnosed with ROP zone 1, zone 2, or AP-ROP (Table 1).

**Table 1:** Characteristics of included studies.

Author	Design	Country	Follow-up time	N° of participants (N° eyes)	Gestational age (weeks) (Mean ± SD)	Birth weight (grams) (Mean ± SD)	Male (*n* %)	Population
Kabataş et al. (20)	Retrospective NRSI Open label	Turkey	More than 18 months	Total: 128 (256)				Type 1 ROP
				1. Bevacizumab (0.625 mg): 12 (24)	1. Bevacizumab (0.625 mg): 26.1 ± 2.27	1. Bevacizumab (0.625 mg): 841 ± 235	1. Bevacizumab (0.625 mg): 7 (58.33)	
				2. Ranibizumab (0.25 mg): 6 (12)	2. Ranibizumab (0.25 mg): 26 ± 1.26	2. Ranibizumab (0.25 mg): 840 ± 177	2. Ranibizumab (0.25 mg): 3 (50)	
				3. Laser: 36 (72)	3. Laser: 27.7 ± 2.7	3. Laser: 1112 ± 362	3. Laser: 15 (41.67)	
				4. No treatment: 74 (148)	4. No treatment: 30.42 ± 2.1	4. Without Treatment: 1317 ± 331	4.Without Treatment: 41 (55)	
Zhang et al. (21)	RCT Open label	China	6 months	Total: 50 (100)				Infants with binocular Zone II treatment-requiring ROP (ROP with Stage 2+ or 3+ in Zone II)
				1. Ranibizumab (0.3 mg): 25 (50)	1. Ranibizumab (0.3 mg): 28.96 ± 1.59	1. Ranibizumab (0.3 mg): 1220 ± 320	1. Ranibizumab (0.3 mg): 14 (56)	
				2. Laser: 25 (50)	2. Laser: 28.27 ± 1.84	2. Laser: 1060 ± 240	2. Laser: 14 (56)	
Stahl et al. (22)	RCT Open label	Multicenter (26 countries)	6 months	Total: 225 (450)				Birthweight less than 1,500 g and a diagnosis of bilateral ROP zone I stage 1+, 2+, 3, or 3+, or zone II stage 3+, or AP-ROP.
				1. Ranibizumab (0.2 mg): 74 (148)	1. Ranibizumab (0.2 mg): 25 (23–32)^a^	1. Ranibizumab (0.2 mg): 791 ± 244	1. Ranibizumab (0.2 mg): 33 (45)	
				2. Ranibizumab (0.1 mg): 77 (154)	2. Ranibizumab (0.1 mg): 26 (23–32)^a^	2. Ranibizumab (0.1 mg): 886 ± 299	2. Ranibizumab (0.1 mg): 37 (48)	
				3. Laser: 74 (148)	3. Laser: 26 (23–32)^a^	3. Laser: 831 ± 284	3. Laser: 37 (50)	
Fleck et al. (23)	RCT Open label	Multicenter (26 countries)	24 months	Total: 180 (360)	Not specified	Not specified	Not specified	Birthweight less than 1,500 g and a diagnosis of bilateral ROP zone I stage 1+, 2+, 3, or 3+, or zone II stage 3+, or AP-ROP.
				1. Ranibizumab (0.2 mg): 61 (122)				
				2. Ranibizumab (0.1 mg): 65 (130)				
				3. Laser: 54 (108)				
Marlow et al. (24)	RCT Open label	Multicenter (26 countries)	24 months	Total: 153 (306)				All study examinations were administered as close as possible to 2 years of age corrected for preterm birth, and data obtained between 20 and 28 months corrected age are included in this article.
				1. Ranibizumab (0.2 mg)2: 56 (112)		1. Ranibizumab (0.2 mg): 10.6 (1.6)		
				2. Ranibizumab (0.1 mg): 53 (106)		2. Ranibizumab (0.1 mg): 10.3 (2.0)		
				3. Laser: 44 (88)	Not specified	3. Laser: 10.8 (1.7)	Not specified	
Fidler et al. (25)	RCT Open label	Multicenter (26 countries)	6 months	Total: 218 (436)				Birthweight less than 1,500 g and a diagnosis of bilateral ROP zone I stage 1+, 2+, 3, or 3+, or zone II stage 3+, or AP-ROP.
				1. Ranibizumab (0.2 mg):73 (146)	1. Ranibizumab (0.2 mg): 25.8 (2.25)	1. Ranibizumab (0.2 mg) = 0.79 (0.24)	1. Ranibizumab (0.2 mg) = 40 (54.8)	
				2. Ranibizumab (0.1 mg): 76 (152)	2. Ranibizumab (0.1 mg): 26.5 (2.52)	2. Ranibizumab (0.1 mg) = 0.886 (0.30)	2. Ranibizumab (0.1 mg) = 40 (52.6)	
				3. Laser group: 69 (138)	3. Laser group = 26.2 (2.59)	3. Laser group = 0.83 (0.28)	3. Laser group = 34 (49.3)	

RCT, randomized controlled trial; ROP, retinopathy of prematurity; AP-ROP, aggressive posterior retinopathy of prematurity; SD, standard deviation.

^a^ Median and Interquartile Range (IQR).

### 3.3. Risk of bias

**Figure 2:**
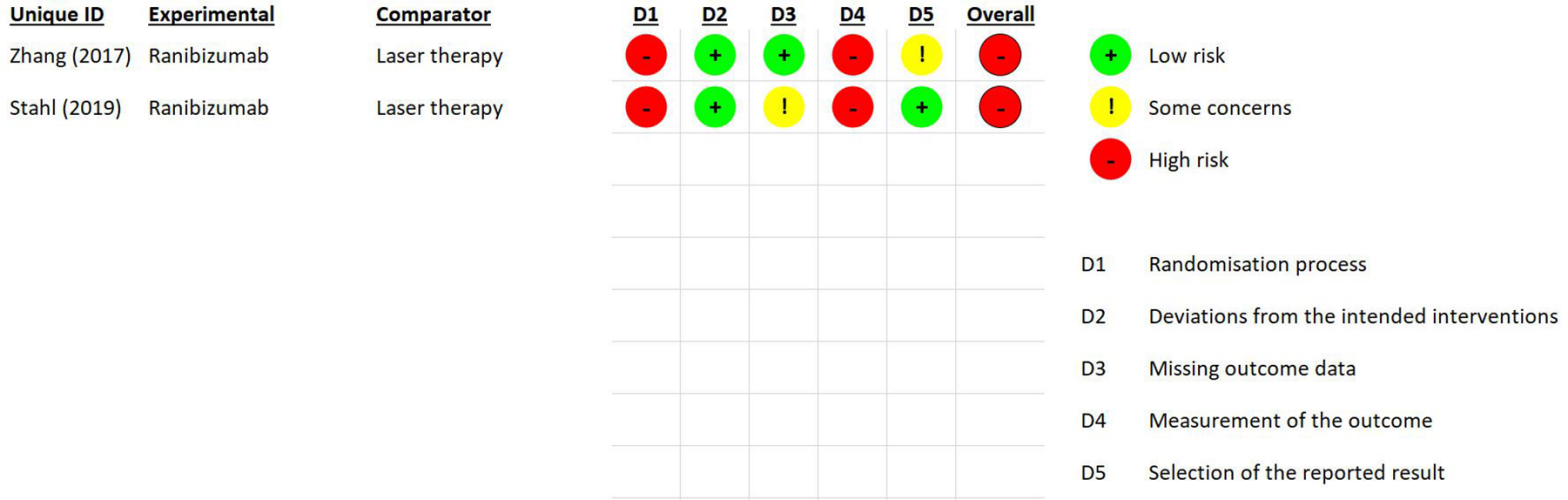
Risk of bias summary.

Using the RoB-2 tool to assess the risk of bias, the studies by Zhang et al. (21) and Stahl et al. (22) received a high-risk score due to the presence of high risk in the randomization process and measurement of the outcome (Figure 2).

### 3.4. Effect of ranibizumab on primary and secondary outcomes

**Figure 3:**
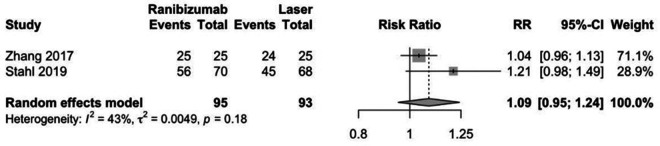
Forest plot of effect of ranibizumab on regression of ROP.

**Figure 4:**
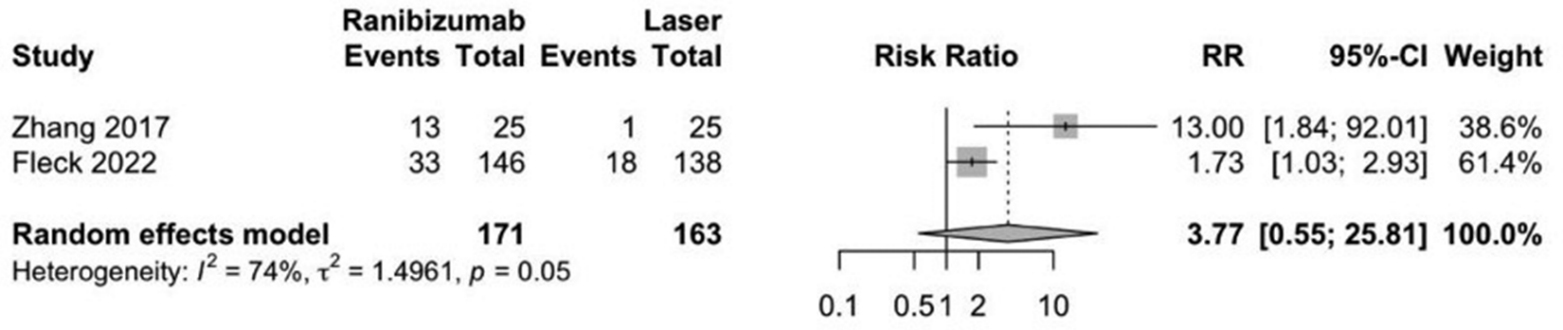
Forest plot of effect of ranibizumab on recurrence of ROP.

Ranibizumab was not associated with greater regression of ROP compared to laser therapy in preterm infants (RR: 1.09, CI 95%: 0.95–1.24; *p*: 0.22; Figure 3). Also, ranibizumab was not associated with recurrence of ROP compared to laser therapy in preterm infants (RR: 3.77, CI 95%: 0.55–25.81; *p*: 0.22; Figure 4).

### 3.5. Adverse effects and/or complications

No serious adverse events or serious complications related to ranibizumab treatment were reported. Table 2 shows the adverse events and/or complications reported in the studies.

**Table 2:** Adverse events and/or complications reported.

Author	Group	Adverse event and/or complications (N° of patients)	Commentary
Kabatas et al. (20)	Bevacizumab (0.625 mg)	Preretinal hemorrhage (2).	The preretinal hemorrhages were reabsorbed 3 weeks later without sequelae in all 3 groups. Exudative retinal detachment resolved 3 weeks later.
	Ranibizumab (0.25 mg)	Preretinal hemorrhage (1).	
	Laser	Preretinal hemorrhage (5), exudative retinal detachment (1), macular ectopia (1).	
	No treatment	–	
Zhang et al. (21)	Ranibizumab (0.3 mg)	Not specified.	The vitreous hemorrhage gradually resolved after ranibizumab injection and the ROP subsided.
	Laser	Plus disease, neovascularization with vitreous and retinal hemorrhage (1).	
Stahl et al. (22)	Ranibizumab (0.2 mg)	Retinal hemorrhage (6), conjunctival hemorrhage (6), moderate cataract (1), and conjunctivitis (1).	The infant with endophthalmitis had a periocular staphylococcal infection 11 days before ranibizumab injection.
	Ranibizumab (0.1 mg)	Retinal hemorrhage (10), vitreous hemorrhage (4), conjunctival hemorrhage (6), conjunctivitis (6), and unilateral endophthalmitis (1).	
	Laser	hemorragia retinal (7), hemorragia conjuntival (2), conjuntivitis (3), and opacidad corneal (2).	
Fleck et al. (23)	Ranibizumab (0.2 mg)	Not specified.	–
	Ranibizumab (0.1 mg)		
	Laser		
Marlow et al. (24)	Ranibizumab (0.2 mg)	High myopia (4).	The investigators observed no late ocular complications or adverse events at 2-year follow-up. In the group that received ranibizumab there was a lower risk of high myopia at 2 years compared to laser.
	Ranibizumab (0.1 mg)	Not specified.	
	Laser	High myopia (14).	
Fidler et al. (25)	Ranibizumab (0.2 mg)	Not specified.	–
	Ranibizumab (0.1 mg)		
	Laser		

ROP, retinopathy of prematurity.

### 3.6. Certainty of evidence

There is a very low level of certainty of evidence for the use of ranibizumab compared to laser in the regression and recurrence of ROP. This is related to the risk domains of bias, imprecision, and inconsistency (Table 3).

**Table 3:** Summary of findings.

Outcome № of participants (studies)	Relative effect (95% CI)	Anticipated absolute effects (95% CI)			Certainty
				Difference	
Regression of ROP (R. ROP) assessed with: Frequency № of participants: 188 (2 RCTs)	**RR: 1.09** (0.95–1.24)	74.2%	**80.9%** (70.5–92)	**6.7% more** (3.7 fewer to 17.8 more)	⊕◯◯◯ Very low
Recurrence of ROP assessed with: Frequency № of participants: 334 (2 RCTs)	**RR: 3.77** (0.55–25.81)	11.7%	**43.9%** (6.4–100)	**32.3% more** (5.2 fewer to 289.2 more)	⊕◯◯◯ Very low

CI, confidence interval; RR, risk ratio.

## 4. Discussion

This systematic review and meta-analysis founded that ranibizumab did not increase the regression of ROP and the recurrence of ROP compared to laser therapy.

In the individual results of the RCTs, we founded a few things that were at odds with our overall conclusions. For example, in the study by Fleck et al. (2022), it was stated that ROP decreased significantly faster after an intravitreal injection of ranibizumab than when there was more disease, stage 3 ROP, or ROP-AP (aggressive posterior), compared to laser treatment (23).

The study by Stahl A. et al. discovered that treatment success occurred in 56 (80%) of 70 infants receiving ranibizumab 0–2 mg compared with 57 (75%) of 76 infants receiving ranibizumab 0–1 mg and 45 (66%) of 68 infants following laser therapy, despite the fact that few studies have found specific treatment-related outcomes (22).

ROP is a medical disorder that impacts the retinal development of preterm infants (26). It is caused by abnormal proliferation of blood vessels in the retina, which can result in scarring, retinal detachment, and visual loss. ROP is a dangerous disorder that, if left untreated, can result in irreversible visual impairment or blindness (27).

Infants born extremely early or with an extremely low birth weight have the highest chance of developing ROP. Laser surgery or other methods may be used to halt the aberrant blood vessel development and avoid additional retinal damage (28).

The BEAT-ROP (bevacizumab) (29) and RAINBOW (ranibizumab) (24) trials reported a lower prevalence of very high and high myopia, respectively, compared with laser therapy. The proposed mechanisms that explain why laser therapy increases the risk of high myopia are controversial (30). It was previously reported that intravitreal bevacizumab (anti-VEGF) allows retinal vessel development beyond the neovascular ridges (31), which is minimal after laser use. This event probably allows the expression of signaling pathways necessary for the development of the anterior segment with minimal myopia (29).

The specific etiology of ROP is unknown, however it is believed to be associated with aberrant blood vessel proliferation in preterm newborns’ retinas (32).

The VEGF is a protein that is involved in the pathophysiology of ROP and plays a vital function in the formation of blood vessels (33).

Under normal conditions, VEGF production is tightly controlled to enable healthy development of blood vessels in the retina (34).

However, in preterm infants, the retinal blood vessels may not be fully grown, making them more susceptible to fluctuations in oxygen levels. In an attempt to accelerate the creation of new blood vessels, cells in the retina may release excessive levels of VEGF when the infant encounters hypoxia (35).

Retinal cells generate VEGF, which encourages the formation of new blood vessels. Under normal conditions, VEGF production is tightly controlled to enable healthy development of blood vessels in the retina (36).

In preterm infants, however, VEGF synthesis may be aberrant, resulting in faulty blood vessel development in the retina. This can lead to the development of ROP, which can result in retinal scarring and detachment and visual loss (37).

The dysregulation of VEGF production in response to hypoxia (oxygen deficiency) is believed to have a major role in the development of aberrant blood vessel growth and neovascularization in ROP (38).

VEGF inhibitors, such as ranibizumab, have been utilized to treat ROP by reducing the synthesis of VEGF and halting the aberrant development of blood vessels (39).

Bevacizumab has also been studied as a treatment for ROP with encouraging results (29, 31). However, in preterm infants, use of bevacizumab showed a serum half-life of 21 days and persistent detectable levels after 60 days (40). On the other hand, ranibizumab showed a much shorter serum half-life (5.6 days) and greatly decreased serum levels at 28 days (25). Adequate ocular retention and rapid systemic excretion of ranibizumab may provide satisfactory ocular efficacy and a favorable systemic safety profile (25).

## 5. Conclusions

The evidence for the efficacy of ranibizumab compared to laser is very uncertain in terms of ROP regression and decreased ROP recurrence in preterm infants. Although individual evidence may support the efficacy of ranibizumab, the differences between studies do not allow us to generalize in its favor. Consideration should be given to analyzing more findings where comparisons are less heterogeneous, so as to ensure quality and certainty of evidence.

## Data Availability

The original contributions presented in the study are included in the article/Supplementary Material, further inquiries can be directed to the corresponding author.
